# An extragenic second-site mutation in the *jar1-1* mutant suppresses the response to photoperiod stress independent of jasmonic acid

**DOI:** 10.1007/s11103-025-01602-9

**Published:** 2025-06-29

**Authors:** Anne Cortleven, Silvia Nitschke, Venja Roeber-Terstegen, Cornelia Herrfurth, Ivo Feussner, Thomas Schmülling

**Affiliations:** 1 Institute of Biology, Applied Genetics, Dahlem Centre of Plant Sciences (DCPS), Freie Universität Berlin, Albrecht-Thaer-Weg 6, 14195 Berlin, Germany; 2 Department of Plant Biochemistry, Albrecht-Von-Haller-Institute for Plant Sciences and Göttingen Center of Molecular Biosciences (GZMB), University of Göttingen, Justus-Von-Liebig-Weg 11, 37077 Göttingen, Germany; 3 Service Unit for Metabolomics and Lipidomics, Göttingen Center of Molecular Biosciences (GZMB), University of Göttingen, Justus-Von-Liebig-Weg 11, 37077 Göttingen, Germany; 4 Present Address: Division of Neurology, Department of Pediatrics, University of Texas Southwestern Medical Center, Dallas, TX 75390 USA

**Keywords:** Abiotic stress, *Arabidopsis thaliana*, Circadian clock, Cytokinin, Extragenic suppressor, Hydrogen peroxide, JAR1, Jasmonic acid, Photoperiod, Photoperiod stress

## Abstract

Extension of the light period causes photoperiod stress in *Arabidopsis thaliana*. The photoperiod stress phenotype is characterized by an induction of stress and cell death marker genes, the formation of reactive oxygen species (ROS) and enhanced formation of jasmonates during the night following the extended light period. Previously, experiments had shown that the *jar1-1* mutant, carrying a point mutation in the jasmonoyl-isoleucine (JA-Ile) biosynthesis gene *JAR1*, showed a strongly reduced stress phenotype suggesting that JA-Ile is required for the stress response. Here, we have analyzed the roles of JA-Ile and *JAR1* in more detail. While *jar1-1* reduced the photoperiod stress phenotype indicating that *JAR1* is required for the response to photoperiod stress, mutation of the *ALLENE OXIDE SYNTHETASE* (*AOS*) jasmonate biosynthesis gene did not rescue the stress phenotype. Further, analysis of jasmonate signaling mutants did not indicate their broad resistance to photoperiod stress. Unexpectedly, other *JAR1* mutant alleles like *jar1-11* and *fin219-2* did not alleviate the photoperiod stress phenotype. Genetic analysis revealed that a recessive unlinked second-site mutation in the *jar1-1* mutant background is responsible for the suppression of the photoperiod stress response. Taken together, these results suggest that JA-Ile is less important for the response to photoperiod stress than indicated by previous results.

**Supplementary Information:**

The online version contains supplementary material available at 10.1007/s11103-025-01602-9.

## Key message

An extragenic second-site mutation in the *jar1-1* mutant with an unknown function is important for the response to photoperiod stress and acts independently of jasmonic acid.

**Supplementary Information:**

The online version contains supplementary material available at 10.1007/s11103-025-01602-9.

## Introduction

Numerous developmental processes in plants are controlled by the photoperiod (Jackson 2009). As the Earth turns around its own axis, the resulting change of day and night causes the need to adapt life processes to this daily rhythm. Recently, it was discovered that the extension of the light period causes stress in plants, which has been named photoperiod stress (originally circadian stress) (Nitschke et al. 2016, 2017). Hallmarks of the stress phenotype are the induction of stress marker genes such as *RESPONSIVE TO HIGH LIGHT41* (*ZAT12*) and *BON ASSOCIATION PROTEIN1* (*BAP1*) and an increase in oxidative stress (peroxides) during the night following the prolonged light period (Nitschke et al. 2016; Abuelsoud et al. 2020; see Roeber et al. 2022, for a review). The next day, a significant reduction of PSII maximum quantum efficiency (F_v_/F_m_) and lesion formation in the leaves may ensue in photoperiod stress-sensitive genotypes (Nitschke et al. 2016). An extension of the light period by one hour in short day-adapted plants was sufficient to provoke a stress response in wild-type plants (WT) (Abuelsoud et al. 2020; Roeber-Terstegen 2024) and both chloroplast-dependent and photoreceptor-dependent signaling is required to induce photoperiod stress (Bajaj Hengge et al. 2025).

Different plant hormones are involved in the response to photoperiod stress. Cytokinin (CK), and in particular root-derived *trans*-zeatin, protects against photoperiod stress while auxin promotes it (Frank et al. 2020, 2022). The concentrations of the stress hormones salicylic acid and jasmonic acid (JA) increase during the night following an extended light period. Moreover, numerous genes involved in JA biosynthesis and JA signaling have been found to be responsive to photoperiod stress (Nitschke et al. 2016; Cortleven et al. 2022). This and the fact that plants carrying the *jar1-1* mutation showed a reduced stress response, in particular in CK-deficient plants, led to the proposal that the increase in jasmonates may be causal for the photoperiod stress response (Nitschke et al. 2016).

It is known that jasmonates accumulate rapidly in response to mechanical damage or herbivory resulting in the activation of defense gene expression (reviewed by Howe et al. 2018; Wasternack and Feussner 2018; Wasternack and Hause 2013). Jasmonates are lipid-derived molecules which are generated via one specific branch of oxylipin biosynthesis, the AOS branch (Wasternack and Kombrink 2010). Biosynthesis of JA starts with the dioxygenation of α-linolenic acid (18:3) or roughanic acid (16:3). Both are trienoic fatty acids (TFA) which are released from plastidial galactolipids (Wallis and Browse 2002). In *Arabidopsis* three ω-3 fatty acid desaturases (FAD3, FAD7, and FAD8) mediate the desaturation of 16:2 and 18:2 fatty acids to 16:3 and 18:3 (McConn and Browse 1996). After its release from the membrane, the free TFA α-linolenic acid is converted by a lipoxygenase to 13-hydroperoxylinolenic acid. Subsequent enzymatic reactions catalyzed by allene oxide synthase (AOS) and allene oxide cyclase (AOC) result in the formation of 12-oxo-phytodienoic acid (OPDA). The action of OPDA reductase 3 followed by three cycles of β-oxidation yields JA which is metabolized by an ATP-dependent JA-amino synthetase (Staswick et al. 2002; Staswick and Tirayaki 2004), encoded by *JASMONATE RESISTANT1* (*JAR1*), into the biologically active JA-Ile. In the absence of JA-Ile, JAZMONATE ZIM DOMAIN (JAZ) proteins bind and repress JA-Ile-dependent transcription factors such as MYC2, MYC3, MYC4 by recruiting the co-repressors TOPLESS (TPL) and TPL-related (TRP) proteins through interaction with NOVEL INTERACTOR OF JAZ (NINJA) (Pauwels et al. 2010). TPL recruits histone deacetylases and methyltransferases to inhibit transcription (Wang et al. 2013). In presence of JA-Ile, the receptor and F-box protein CORONATINE-INSENSITIVE1 (COI1) of the E3 ubiquitin ligase SCF^COI1^ (Xie et al. 1998) interacts with JAZ proteins. This interaction mediates ubiquitination and degradation of JAZs resulting in the release of transcription factors (MYC2, MYC3 and MYC4) and expression of JA-Ile response genes (Chini et al. 2007; Sheard et al. 2010), including JA biosynthesis genes (Thines et al. 2007; Chung et al 2008; Wasternack and Hause 2013). Besides this MYC branch of JA signaling, also an ETHYLENE RESPONSE FACTOR1 (ERF1) branch exists (Pré et al. 2008). The ERF1 branch requires both JA-Ile and ethylene signaling and involves the ethylene-stabilized transcription factors ETHYLENE-INSENSITIVE 3/EIN3-LIKE1 (EIN3/EIL1) which act upstream of specific APETALA/ETHYLENE RESPONSE FACTOR (ERF) transcription factors like ERF1 and OCTADECANOID RESPONSIVE ARABIDOPSIS59 (ORA59).

CK is another plant hormone that regulates numerous developmental processes such as the cell cycle, shoot and root growth, leaf senescence and seed germination but has also diverse functions in response to abiotic and biotic stress (Werner and Schmülling 2009; Cortleven et al. 2019). For example, CK protects plants from high light stress (Cortleven et al. 2014) and induces resistance against pathogen attack (Choi et al. 2010). The CK signal is perceived by three membrane-localized sensor histidine kinases, ARABIDOPSIS HISTIDINE KINASE2 (AHK2), AHK3 and CYTOKININ RESPONSE1/AHK4 (CRE1/AHK4) (Inoue et al. 2001; Suzuki et al. 2001), which have both distinct and overlapping functions (Higuchi et al. 2004; Nishimura et al. 2004; Riefler et al. 2006; Heyl et al. 2012). The CK signal is further submitted through a phosphorelay to type-B response regulators which regulate the transcription of cytokinin response genes (Kieber and Schaller 2014). In the response to photoperiod stress CK acts through the receptor AHK3 and the type-B ARABIDOPSIS RESPONSE REGULATOR2 (ARR2), ARR10 and ARR12 (Nitschke et al. 2016; Frank et al. 2020).

In this study we have investigated the putative role of JA-Ile and *JAR1* in the response to photoperiod stress in more detail. Neither biochemical analysis of precursors of JA biosynthesis nor the stress response of mutants of JA biosynthesis yielded additional indication for a role of JA in the photoperiod stress response. Analysis of the photoperiod stress response in additional *JAR1* mutant alleles like *jar1-11* and *fin219-2* revealed no strong reduction of the stress response in comparison to WT suggesting that not *JAR1* but probably a second-site mutation in *jar1-1* causes the reversion of the photoperiod stress phenotype. This was confirmed by genetic analysis identifying a recessive mutation in a locus independent of *jar1-1*, which we named *SUPPRESSOR OF PHOTOPERIOD STRESS* (*SOPS*). The mutation of *SOPS* suppressed the photoperiod stress response in WT as well as in the sensitive *ahk2 ahk3* cytokinin receptor mutant background. The identification of the yet unknown *SOPS* gene will help to elucidate the mechanism of the photoperiod stress response.

## Material and methods

### Plant material and growth conditions

*Arabidopsis thaliana* accession Col-0 was used as WT with exception of the control for the *opr3* mutant which is in the Wassilewskija (Ws) background. The following mutant and transgenic plants were used: *35S::CKX4* (Werner et al. 2003), *ahk2-5 ahk3-7* (Riefler et al. 2006), *jar1-1* (Staswick et al. 2002), *jar1-11* (SALK_034543), *fin219-2* (SALK_059774), *jar1-1 ahk2-5 ahk3-7*, *jar1-1 Pro35S:CKX4* (Nitschke et al. 2016), *fad3-2 fad7-2 fad8* (McConn and Browse 1996), *aos* (SALK_017756), *dde2-2* (von Malek et al. 2002), *lox3* (SALK_062064), *lox4* (SALK_071732), *opr3* (in Ws background) (Stinzi and Browse 2000), *coi1-t* (SALK_035548)(Mosblech et al. 2011), *ora59* (GABI_061A12), and *myc2 myc3 myc4* (Fernandez-Calvo et al. 2011). Seeds were obtained from the European Arabidopsis Stock Centre (NASC; http://arabidopsis.info/) or kindly provided by Rachel Green, Alain Goossens, and John Browse. The following mutants were generated in this work by genetic crosses: *aos ahk2-5 ahk3-*7, *dde2-2 ahk2-5 ahk3-7*, *myc3 myc4 ahk2-5 ahk3-*7, *ora59 ahk2-5 ahk3-7*, *coi1-t ahk2-5 ahk3-7,* and *jar1-11 ahk2 ahk3*. The genotype of multiple mutants was confirmed by PCR-based analysis.

*Arabidopsis* plants were grown on soil under short day (SD) conditions (8 h light/16 h darkness) in a growth chamber, light intensities of 100 to 150 µmol m^−2^ s^−1^, using a combination of Philips Son-T Agros 400W, and Philips Master HPI-T Plus, 400W/645 lamps, at 22 °C and 60% relative humidity. *coi1* and *coi1 ahk2 ahk3* plants were first grown for two weeks under SD conditions on MS plates containing 25 µM methyl jasmonate. Surviving mutants (homozygote) were transferred to soil and grown until they were five weeks old under the conditions described above.

### Stress treatments

**Fig. 1:**
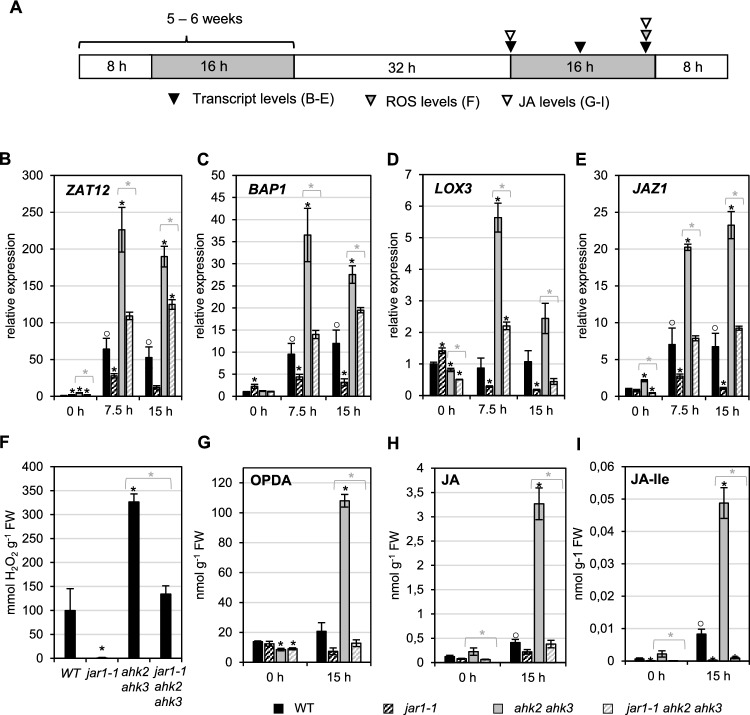
The *jar1-1* mutation causes a reduced response to photoperiod stress in wild type and stress-sensitive *ahk2 ahk3* cytokinin receptor mutants. **A** Schematic overview of experimental conditions used in (**B**–**I**). Plants were grown under short day conditions for five weeks before exposure to a prolonged 32-h light period (PL). Leaf samples were collected at the indicated time points (triangles). White, light period; grey, dark period. **B**–**E** Transcript levels of oxidative stress marker genes *ZAT12* (**B**) and *BAP1* (**C**), JA biosynthesis gene *LOX3* (**D**) and JA signaling gene *JAZ1* (**E**) at the onset (0 h) and during the dark period at 7.5 h and 15 h after PL treatment. Transcript levels of WT at 0 h were set to 1. Data are mean values ± SE (n = 4). Asterisks indicate significant differences of mutants from WT (black) and between cytokinin-deficient plants in WT or *jar1-1* background (grey). Symbol ○ indicates significant differences between WT at 0 h and other time points (for WT only) (p < 0.05). F, H_2_O_2_ levels measured during the night at 15 h after PL treatment (mean values ± SE; n = 5). G-I, JA metabolite levels in the dark period (0 h and15 h) following PL treatment. Data are mean values ± SE (n = 5). Asterisks indicate significant differences from WT (black) and between cytokinin-deficient plants in WT or *jar1-1* background (grey) (p < 0.05). FW, fresh weight

For stress treatments, five to six weeks old SD-grown plants were used. The standard stress regime consisted of a 32 h light treatment (prolonged light period, PL) integrated into a SD regime (Fig. 1A). Control plants remained under SD condition. For phenotypical analyses, affected and unaffected leaves from stress-treated plants of the same developmental stage were chosen. For RNA and H_2_O_2_ measurements, only the distal parts of these leaves were harvested, which is the most affected part of the leaves. Whole leaves were used for F_v_/F_m_ measurements. Harvest during the dark period was performed in green light.

### Analysis of cell death

Mature leaves, defined as fully expanded leaves, with lesions were counted 20 to 24 h after the prolonged light treatment. Percentage of lesions refers to the percentage of mature leaves with lesions.

### Fluorometry

Chlorophyll fluorescence emission was measured on detached leaves with a modulated chlorophyll fluorometer (Photosystem Instruments, Drasov, Czech Republic). After dark adaptation for 20 min, the maximal photochemical efficiency of PSII was determined from the ratio of variable (F_V_) to maximum (F_M_) fluorescence [F_V_/F_M_ = (F_M_-F_0_)/F_M_]. An actinic light pulse (0.2 µmol m^−2^ s^−1^) was used to determine the initial (minimum) PSII fluorescence in the dark-adapted state (F_0_), and F_M_ was determined by a saturating light pulse (1.500 µmol mol^−2^ s^−1^).

### Determination of peroxides

Leaf material (leaves 7–10) was flash frozen and homogenized using a Retsch Mixer Mill MM2000 (Retsch, Haan, Germany) with two stainless-steel beads (2 mm diameter) in each sample. H_2_O_2_ levels were determined using Pierce™ Quantitative Peroxide Assay Kit (Aqueous) (ThermoFischer Scientific, Berlin, Germany) as described by Abuelsoud et al. 2020.

### RNA isolation and quantitative RT-PCR

RNA isolation from leaves and quantitative RT-PCR were carried out according to Frank et al. (2020). In brief, leaf material (leaves 7 -10) was harvested, flash-frozen and homogenized with a Retsch Mixer Mill MM2000 (Retsch, Haan, Germany) with two stainless-steel beads (2 mm diameter) in each sample. Total RNA was extracted from entire seedlings using the NucleoSpin® RNA plant kit (Machery and Nagel, Düren, Germany) as described in the user’s manual or by a phenol/chloroform/LiCl isolation adapted from Sambrook and Russell (2001). After DNAse treatment (Fermentas, Life Technologies, Darmstadt, Germany), equal amounts of starting material (1 µg RNA) were used in a 20 µl SuperScript® III Reverse Transcriptase reaction. Primer pairs were designed using Primer 3 Software (http://www.genome.wi.mit.edu/cgibin/primer/primer3.cgi) or Quantprime Software (Arvidsson et al. 2008). Primers used for reference genes and genes of interest are listed in Supplemental Table S1. Real-time PCR using FAST SYBR Green I technology was performed on an CFX96 Touch Real-Time Detection System (Biorad, Feldkirchen, Germany) and the following cycling conditions (15 min 95 °C, 40 cycles of 5 s at 95 °C, and 15 s at 55 °C and 10 s at 72 °C), followed by the generation of a dissociation curve to check for specificity of the amplification. Reactions contained SYBR Green Master Mix, Immolase (Bioline, Memphis, USA), 300 nM of a gene specific forward and reverse primer and 2 µl of a 1:10 diluted cDNA in a 20 µl reaction. Gene expression data were normalized against two or three different nuclear-encoded reference genes (*UBC21, PP2A* and/or *MCP2A*) according to Vandesompele et al. (2002) and presented relative to WT.

### Analysis of jasmonates

Extraction and analysis of JAs by UPLC-nano ESI–MS/MS was performed as recently described with some modifications (Kusch et al. 2019): 214/62 [declustering potential (DP) -35 V, entrance potential (EP) -8.5 V, collision energy (CE) -24 V] for D_5_-JA, 209/59 (DP -30 V, EP -4.5 V, CE -24 V) for JA, 265/151 (DP -80 V, EP -10 V, CE -35 V) for OPC6, 296/170 (DP -70 V, EP -8.5 V, CE -28 V) for D_5_-oPDA, 291/165 (DP -70 V, EP -8.5 V, CE -28 V) for OPDA, 325/133 (DP -65 V, EP -4 V, CE -30 V) for D_4_-JA-Leu, and 322/130 (DP -45 V, EP -5 V, CE -28 V) for JA-Ile. The mass analyzers were adjusted to a resolution of 0.7 amu full width at half-height. The ion source temperature was 40 °C, and the curtain gas was set at 10 (given in arbitrary units). Quantification was carried out using a calibration curve of intensity (*m/z*) ratios of [unlabeled]/[deuterium-labeled] *vs.* molar amounts of unlabeled (0.3–1000 pmol). Peak areas were collected with the Analyst software (AB Sciex, Framingham, MA, USA).

### Determination of free fatty acids

For quantitative determination of free fatty acids by gas chromatography equipped with a flame ionization detector (GC-FID) plant material (200 mg) was extracted by adding 4 mL of extraction medium (n-hexane: 2-propanol, 3:2 [v/v] with 0.0025% [w/v] butylated hydroxytoluene) and 2 µg pentadecanoic acid as internal standard. The extract was shaken for 10 min and centrifuged at 625 × *g* at 4 °C for 10 min. The upper phase was collected, and a 6.7% (w/v) solution of potassium sulfate was added up to a volume of 6.5 ml. After vigorous shaking and centrifugation at 625 × *g* at 4 °C for 10 min the upper hexane-rich layer was subsequently dried under streaming nitrogen. For preparation of methyl esters of fatty acids (FAMEs) for analysis by gas chromatography-flame ionization detection (GC-FID), the sample was redissolved in 400 µL of methanol and 6.5 µL of trimethylsilyldiazomethane (2 M in hexane; Sigma) were added. After shaking for 30 min, 0.2 µL of glacial acetic acid were added. The resulting FAMEs were dried under streaming nitrogen and redissolved in 20 µL acetonitrile. The analysis of the FAME was performed with an Agilent (Waldbronn, Germany) 6890 gas chromatograph fitted with a capillary DB-23 column (30 m × 0.25 mm, 0.25 µm coating thickness; J&W Scientific, Agilent). Helium was used as carrier gas (1 mL min^−1^). The temperature gradient was 150 °C for 1 min, 150–200 °C at 8 K min^−1^, 200–250 °C at 25 K min^−1^ and 250 °C for 6 min. Peak areas were collected with the ChemStation software (Agilent, Waldbronn, Germany).

### Statistical analysis

Statistical analyses were performed using SAS v.9.2 (SAS Institute GmbH, Heidelberg, Germany). Data were analyzed by a one-way ANOVA followed by Tukey’s post hoc test. Normality and homogeneity of variance were tested using the Shapiro–Wilk and Levene’s tests (Neter et al. 1996). To meet the assumptions, datasets were transformed using log or square root transformation. When assumptions were not met, a non-parametric Kruskal–Wallis test was performed followed by a Wilcoxon-Mann–Whitney test to perform pair-wise comparisons.

## RESULTS

### Introgression of *jar1-1* in cytokinin-deficient plants alleviates the photoperiod stress phenotype

Introgression of *jar1-1* into plants with lowered CK-content or signaling partially rescued the strong photoperiod stress phenotype by reducing the number of leaf lesions and the negative impact on photosynthetic capacity (Nitschke et al. 2016) (Supplemental Fig. S1A-B). To investigate the impact of the *jar1-1* mutation on the molecular level, the expression of stress marker genes (*ZAT12, BAP1*) and of JA-Ile-responsive genes (*LOX3, JAZ1*) was measured in CK-deficient plants with and without the *jar1-1* mutation and compared to the expression in WT and *jar1-1*. Total RNA was extracted from five-week-old short-day (SD)-grown plants at the beginning, in the middle and close to the end of the night following a prolonged light period (PL) (Fig. 1A). In WT the expression levels of *ZAT12, BAP1* and *JAZ1* increased in response to photoperiod stress (Fig. 1B-E). The response to photoperiod stress was lower in the *jar1-1* mutant than in WT. A much stronger induction of all four marker genes was observed in both the *ahk2 ahk3* receptor mutant (Fig. 1B-E) and *35S:CKX4* plants, which overexpress a CK-degrading *CYTOKININ OXIDASE/DEHYDROGENASE (CKX*) gene (Supplemental Fig. S1C-F). For CK-deficient plants, the strong induction of the marker genes correlated with a strong lesion formation (Supplemental Fig S1A) and a strongly reduced F_v_/F_m_ (Supplemental Fig. S1B). Introgression of *jar1-1* in CK-deficient background significantly decreased the induction of the marker genes (Fig. 1B-E and Supplemental Fig. S1C-F) and also lesion formation (Supplemental Fig. 1A-B) (Nitschke et al. 2016).

Measurement of peroxide levels at the end of the night confirmed the importance of *JAR1* in the stress response as peroxide formation was almost completely absent in the *jar1-1* mutant (Fig. 1F). Induction of peroxides was approximately three-fold higher in *ahk2 ahk3* mutants as compared to WT and introgression of *jar1-1* resulted in a strong inhibition of peroxide accumulation. This showed that *JAR1* is required for formation of peroxides in response to photoperiod stress in both WT and *ahk2 ahk3*.

Measurement of pathway metabolite concentrations (OPDA, JA and JA-Ile) showed that these were strongly enhanced at the end of the night following the extended light period in both WT and *ahk2 ahk3* mutants, the increase being stronger in the latter (Fig. 1G-I). As expected, the plants harboring the *jar1-1* allele showed a strong decrease of the JA-Ile concentration and a lack of increase after photoperiod stress (Fig. 1I). The lack of JA-Ile causing a lack of feed-forward regulation of JA biosynthesis (Wasternack and Hause 2013) might be the reason for a slightly lower JA content in the *jar1-1* and *jar1-1 ahk2 ahk3* mutants (Fig. 1H). Consistent with a missing feed-forward regulation by JA-Ile, in the *jar1-1* mutant background also the concentrations of OPDA and JA did not increase following photoperiod stress (Fig. 1G-H). These results are in accordance with Nitschke et al. (2016) and clearly correlate the induction of stress marker genes and ROS formation with the detrimental consequences for photosynthesis and cell viability. Moreover, they clearly point to a role of *JAR1* in the stress response as stress marker gene induction and ROS levels were reduced in the *jar1-1* background (Fig. 1B-F).

### The JA precursors TFA are not involved in the response to photoperiod stress

**Fig. 2:**
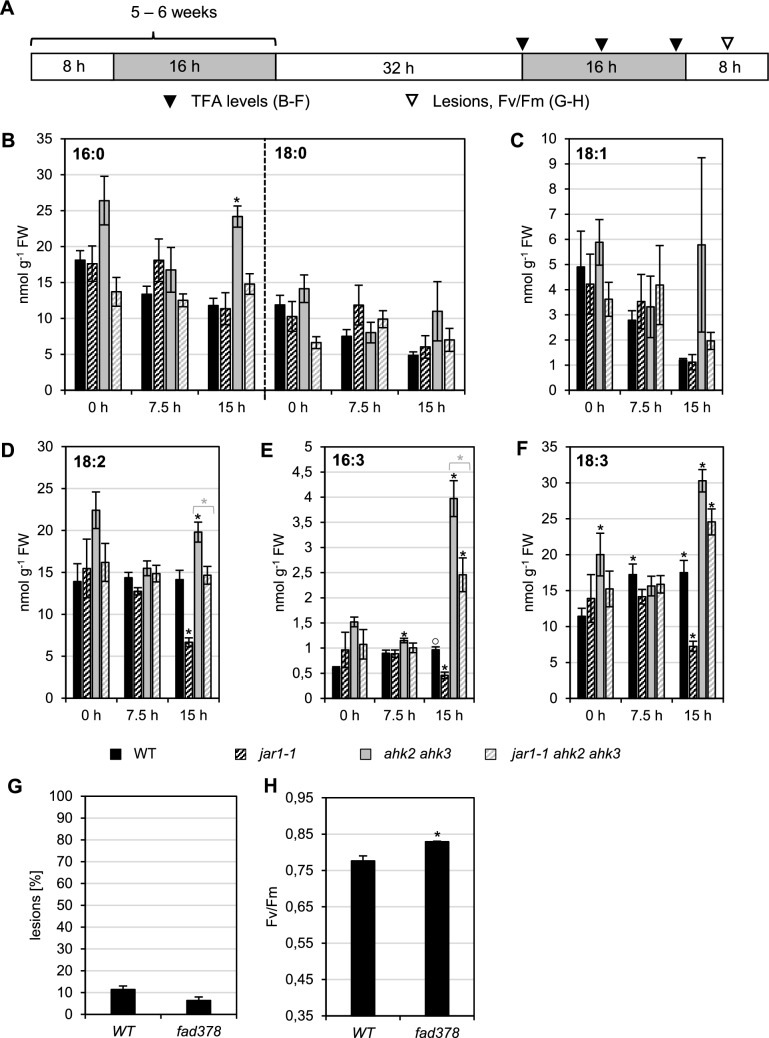
The response to photoperiod stress is independent of free TFAs. **A** Schematic overview of experimental conditions used in B-H. Plants were grown under short day conditions for five weeks before exposure to a prolonged 32-h light period (PL). Leaf samples were collected at the indicated time points (triangles). White, light period; grey, dark period. Free TFA levels (**B**–**F**) were measured at the onset (0 h) and during the dark period at 7.5 h and 15 h after PL treatment. Data are mean values ± SE (n = 5). **G** Percentage of mature leaves with lesions (n ≥ 12). **H** PSII maximum quantum efficiency (Fv/Fm) in representative leaves measured around midday following PL treatment (n ≥ 12). Asterisks indicate significant differences from WT (black) and between cytokinin receptor mutants (*ahk2 ahk3*) in WT or *jar1-1* background (grey). Symbol ○ indicates significant differences between WT at 0 h and the other time points (for WT only) (p < 0.05)

JA biosynthesis starts either with α-linolenic acid or roughanic acid, respectively, after their release from the membrane. To evaluate if photoperiod stress influences the levels of free TFAs, their concentrations were determined at specific time points during the night following the prolonged light period in WT, *jar1-1*, *ahk2 ahk3* and *ahk2 ahk3 jar1-1* (Fig. 2A-F). No differences were observed for 18:0 and 18:1 in none of the genotypes at the different time points (Fig. 2B and 2C). In contrast, the concentrations of 16:0, 16:3 and 18:3 (palmitic acid, roughanic acid and α-linolenic acid) increased 15 h after the end of the prolonged light treatment in *ahk2 ahk3* mutants by about 2–fourfold in comparison to WT. This increase was lower upon introgression of *jar1-1*, while 16:3 and 18:3 contents were below WT levels in the *jar1-1* single mutant 15 h after the end of the prolonged light period (Fig. 2E and 2F). Similar but less strong changes were noted for 18:2 (Fig. 2D). 16:3 and 18:3 can be metabolized for JA biosynthesis and are highly reactive targets for ROS. Transcript levels of the *FATTY ACID DESATURASE3* (*FAD3*), *FAD7* and *FAD8* genes, involved in the transition of linoleic acid (18:2) to α-linolenic acid (18:3) and hexadecadienoic acid (16:2) to roughanic acid (16:3), were not altered in *ahk2 ahk3* mutants in comparison to WT (A.C., I.F. and T.S., unpublished data). The *fad378* triple mutant which is both TFA- and JA-deficient did not have an altered sensitivity to photoperiod stress as compared to WT (Fig. 2G-H). These results argued against a specific role for TFAs in the stress response and questioned the role of jasmonates as TFAs are their metabolic precursors.

### JA biosynthesis does not suppress the response to photoperiod stress

**Fig. 3:**
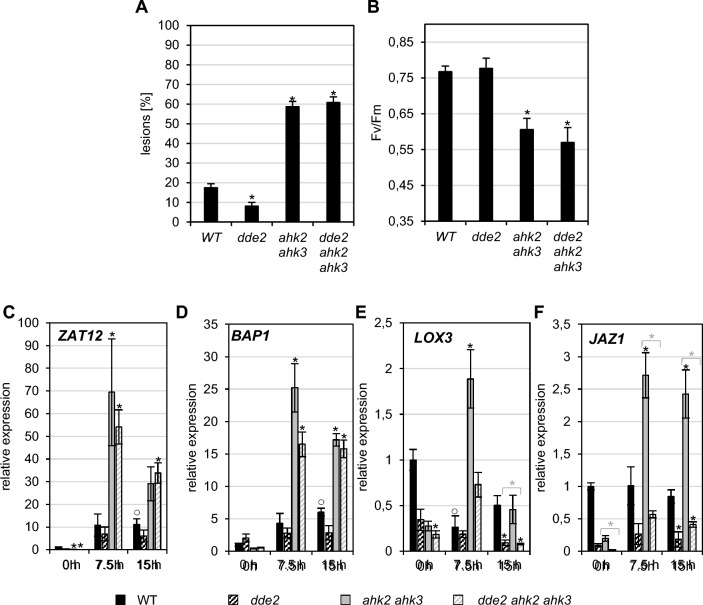
A reduced JA biosynthesis does not rescue the photoperiod stress phenotype in cytokinin receptor mutants. Plants were grown under short day conditions for five weeks before exposure to a prolonged 32-h light period (PL). **A** Percentage of mature leaves with lesions (n ≥ 12). **B** PSII maximum quantum efficiency (F_v_/F_m_) in representative leaves (n ≥ 12) measured around midday following PL treatment. C, H_2_O_2_ levels measured during the night at 15 h after PL treatment (n = 5 ± SE). **D**–**G** Transcript levels of oxidative stress marker genes *ZAT12* (**D**) and *BAP1* (**E**), JA biosynthesis gene *LOX3* (**F**) and JA signaling gene *JAZ1* (**G**) at the onset (0 h) and during the dark period at 7.5 h and 15 h after PL treatment. Transcript levels of WT 0 h were set to 1. Data are mean values ± SE (n ≥ 3). Asterisks indicate significant differences from WT (black) and between cytokinin-deficient plants in WT or *dde2* background (grey). Symbol ○ indicates significant differences between WT at 0 h and the other time points (for WT only) (p < 0.05)

Additional mutants of JA biosynthesis (*lox3, lox4, lox3 lox4, aos, dde2, opr3*) were investigated for their response to photoperiod stress. None of these mutants showed a strongly altered response to photoperiod stress since lesion formation and the reduction in F_v_/F_m_ were similar as in WT (Supplemental Fig. S2A-B; Fig. 3A-B). We selected the *dde2* mutant for closer analysis (Fig. 3). *dde2* is mutated in the *AOS* gene and is completely deficient in OPDA and JA (von Malek et al. 2002). Overall, the response to photoperiod stress, including the induction of stress marker genes, was comparable between *dde2* and WT, suggesting that JA is not required for the stress response in WT (Fig. 3). The *dde2 ahk2 ahk3* triple mutant showed a similar response to photoperiod stress as *ahk2 ahk3* including lesion formation (Fig. 3A), reduction of F_v_/F_m_ (Fig. 3B), and induction of the marker genes *ZAT12* and *BAP1* (Fig. 3C, D). However, the induction of the JA marker genes *LOX3* and *JAZ1* did not occur in the triple mutant (Fig. 3E, F). This can be explained by lack of JA-Ile, required to induce *LOX3* and *JAZ1* and shows that these genes are activated by a different pathway than *ZAT12* and *BAP1*. Similar results were obtained in the *aos* mutant (Supplemental Fig. S3). Together, these data indicate that neither stress gene induction nor lesion formation or reduction of the photosynthetic capacity in response to photoperiod stress require JA-Ile, in both WT and CK-deficient plants.

### Disruption of JA signaling does not completely rescue the photoperiod stress phenotype

**Fig. 4:**
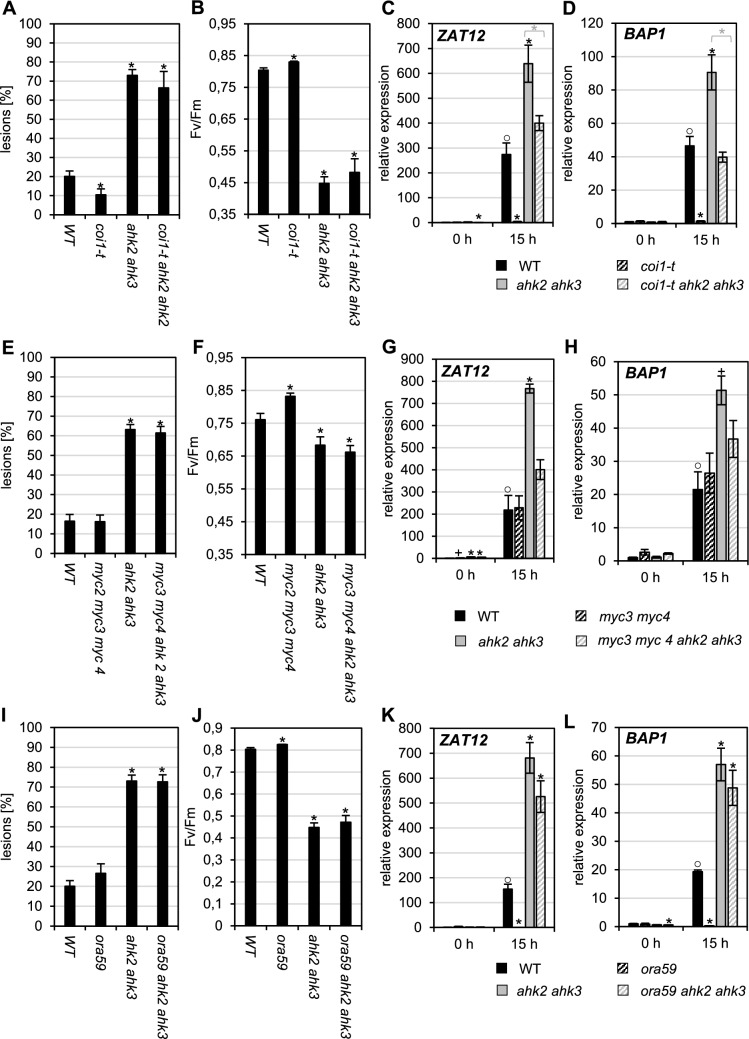
An impaired JA signaling does not completely release the *ahk2 ahk3* mutant from the response to photoperiod stress. Plants were grown under short day conditions for five weeks before exposure to a 32-h light period (PL). **A** Percentage of mature leaves with lesions (n ≥ 12). **B** PSII maximum quantum efficiency (Fv/Fm) in representative leaves measured around midday following PL treatment (n ≥ 12). **C**–**D** H_2_O_2_ levels during the night 15 h after PL treatment. Data are means ± SE (n = 4). E–H. Transcript levels of oxidative stress marker genes *ZAT12* (**E**, **G**) and *BAP1* (**F**, **H**) at the onset (0 h) and during the dark period 15 h after PL treatment. Data are means ± SE (n ≥ 4). Asterisks indicate significant differences from WT (black) and between cytokinin-deficient plants in WT or *coi1/ora59* background (grey). Symbol ○ indicates significant differences between WT at 0 h and the other time points (for WT only) (p < 0.05)

Next, we investigated if a reduced JA-Ile signaling through the COI1 and MYC2 or the ERF branch of JA-Ile signaling affects the photoperiod stress response. In response to photoperiod stress, the *coi1* mutant showed a reduced lesion formation as compared to WT while F_v_/F_m_ was slightly increased (Fig. 4A-B). Strikingly, the induction of stress response genes *ZAT12* and *BAP1* was completely missing in *coi1* (Fig. 4C-D). Introgression of *coi1* into the *ahk2 ahk3* background caused no rescue of the stress phenotype in most experiments, neither for lesion formation nor for photosynthetic efficiency (F_v_/F_m_) (Fig. 4A-B). However, the induction of stress response genes was decreased in *coi1 ahk2 ahk3* compared to *ahk2 ahk3* (Fig. 4C-D). This could be partly due to the recently described broader activity spectrum of COI1 (Ulrich et al. 2021) and needs further clarification.

After the COI1-mediated degradation of the JAZ proteins, several JA-Ile-dependent transcription factors (MYC2, MYC3, MYC4) are activated acting downstream of COI1. Mutation of the corresponding genes did not result in a reduced photoperiod stress phenotype (Fig. 4E-H) indicating that these MYC transcription factors have no role in the response to photoperiod stress in WT. Because of the close linkage of the *MYC2* and *AHK3* genes on chromosome 1 of Arabidopsis we could construct and investigate only the *myc3 myc4 ahk2 ahk3* combination, which showed no significantly different stress response compared to *ahk2 ahk3* (Fig. 4E-H).

Mutation of *ORA59* in the ERF branch of JA signaling did not alter lesion formation or F_v_/F_m_ in response to photoperiod stress, neither in WT nor in *ahk2 ahk3* (Fig. 4I-J). In contrast, the induction of stress marker genes was completely suppressed in the *ora59* mutant (Fig. 4K) while it was only slightly attenuated in the *ora59 ahk2 ahk3* mutant compared to *ahk2 ahk3* (Fig. 4L). Thus, the consequences of the *ORA59* mutation were similar to those of the *COI1* mutation. Together, these results indicate that the ERF branch of JA signaling has no pivotal role for the stress readouts “lesion formation” and “reduction of F_v_/F_m_”, neither in WT nor in the sensitized CK-deficient plants. However, the ERF branch was required for stress marker gene induction in WT but not under stronger stress conditions in *ahk2 ahk3*, indicating that the pathway might be partially involved in the response to photoperiod stress.

### The suppression of the photoperiod stress phenotype is specific for the *jar1-1* mutant

**Fig. 5:**
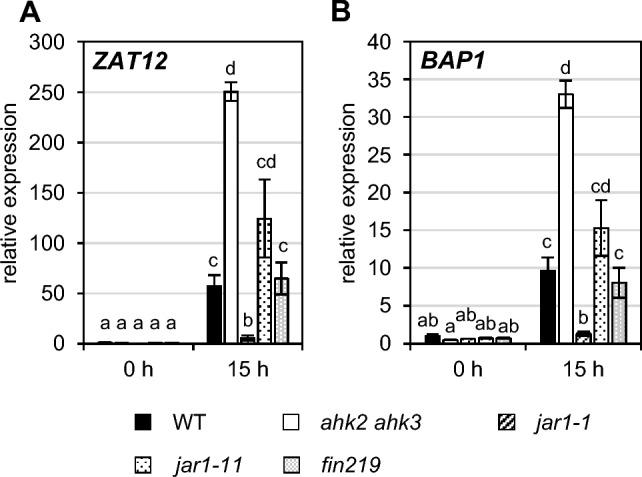
The rescue of the photoperiod stress phenotype is specific for the *jar1-1* mutant. Plants were grown under short day conditions for five weeks before exposure to a 32-h light period (PL). A-B, Transcript levels of oxidative stress marker genes *ZAT12* (**A**) and *BAP1* (**B**) after photoperiod stress in WT and different *jar1* mutants. Transcript levels of WT at 0 h were set to 1. Data are mean values ± SE (n = 4). Letters indicate different statistical groups (p-value ≤ 0.05)

The analysis of JA-related metabolism and testing of JA-related mutants did not support the proposed functional relevance of JA/JA-Ile in response to photoperiod stress. In contrast, these results indicated that JA is less important in mediating photoperiod stress than indicated by the response of the *jar1-1* mutant. It could be, for example, that *JAR1* has an as yet unknown moonlighting function necessary to promote the response to photoperiod stress. It could also be that a second-site mutation in the EMS-induced *jar1-1* mutant (Staswick et al. 1992) reduces stress sensing or the full response to photoperiod stress. Therefore, the photoperiod stress phenotype was evaluated in two other *jar1* mutants, the T-DNA insertion mutants *jar1-11* and *fin219-2* (Wang et al. 2011)*.* The *jar1-1* mutant showed a strongly reduced expression of the stress marker genes *ZAT12* and *BAP1* after photoperiod stress. In contrast, stress marker gene induction was similar to WT in both *jar1-11* and *fin219-2* (Fig. 5A-B). In addition, introgression of *jar1-11* into the photoperiod stress-sensitive *ahk2 ahk3* mutant did not result in an alleviation of the photoperiod stress phenotype as can be seen from stress marker gene expression in these triple mutants after photoperiod stress (Supplemental Fig. S4A-B). This suggests that the suppression of the photoperiod stress phenotype in *jar1-1* and in *jar1-1 ahk2 ahk3* mutants is not caused by the mutation in *JAR1*. Instead, it suggests that a second-site mutation in the EMS-generated *jar1-1* mutant might be causative for the suppressed phenotype.

**Fig. 6:**
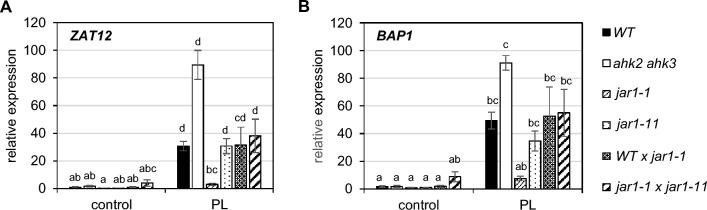
The new trait in *jar1-1* is recessive. Plants were grown under short day conditions for five weeks before exposure to a 32-h light period (PL). Sampling was done one hour before the light switches on. **A**, **B** Transcript levels of photoperiod stress marker genes *ZAT12* (**A**) and *BAP1* (**B**). WT control was set to 1. Data are mean values ± SE (n = 4). Letters indicate different statistical groups (p-value ≤ 0.05)

To investigate whether the resistance trait in the *jar1-1* mutant is recessive or dominant genetic crosses were made between *jar1-1* and WT or *jar1-11* and the response to photoperiod stress was evaluated in the F1 generation. As both the progeny of crosses of *jar1-1* with WT and of *jar1-1* with *jar1-11* showed a photoperiod stress phenotype similar to the stress-sensitive parents we concluded that the novel trait in the *jar1-1* mutant is recessive (Fig. 6). We named the new gene with an as yet unknown identity *SUPPRESSOR OF PHOTOPERIOD STRESS* (*SOPS*).

Next, we studied whether the *sops* mutation is genetically linked to the *jar1-1* locus or segregates independently. These experiments were performed in the stress-sensitive *ahk2 ahk3* background as its strong photoperiod stress phenotype can be easily evaluated. Genetic crosses were made between *jar1-1 ahk2 ahk3* and *ahk2 ahk3*. The resulting F1 plants were self-fertilized to generate an F2 population segregating for *jar1-1* as well as for the *sops* mutation. Out of 96 individuals analyzed of the segregating F2 population, approximately one fourth (19 individuals) showed a photoperiod stress-resistant phenotype like *jar1-1* mutants, which is consistent with the recessive nature of the mutation (Table 1). Of these 19 individuals, three had a homozygote *jar1-1* genotype, 11 individuals were heterozygote (*jar1-1/JAR1*) and 5 were WT (*JAR1/JAR1*). This segregation pattern indicates a single recessive locus that is responsible for the photoperiod stress resistance segregating independently of the *jar1-1* mutation. The independence of the locus is corroborated by the fact that 13 of the F2 plants that were genotyped as homozygote *jar1-1* showed a photoperiod stress response similar to WT. The identification of the *SOPS* gene is beyond the scope of this work but will be a next step in the investigation of photoperiod stress as this gene plays an essential role to sense and/or to respond to this stress.

**Table 1:** Segregation analysis of the progeny of a *jar1-1/JAR1 ahk2 ahk3* mutant plant

Genotypes	Total number of progenies	Photoperiod stress-resistant progenies	Photoperiod stress-sensitive progenies
*JAR1 JAR1*	24	5	19
*jar1-1 JAR1*	56	11	45
*jar1-1 jar1-1*	16	3	13
Total	96	19	77

The parent plant (*jar1-1/JAR1 ahk2 ahk3*) was self-fertilized, plants of the segregating F1 population were genotyped and their response to photoperiod stress was analyzed in five-weeks-old plants

## Discussion

Based on previous results (Nitschke et al. 2016), this work has explored the potential functional relevance of JA/JA-Ile and JAR1 in the response to photoperiod stress. Unexpectedly, this investigation revealed that JA/JA-Ile is not required for a strong response to photoperiod stress. Instead, we identified a second site mutation named *sops* unlinked to the *JAR1* locus as being causal for the suppression of the photoperiod stress response. Therefore, this result corrects the hypothesis that JA/JA-Ile plays a pivotal role in the photoperiod stress response (Nitschke et al. 2016) and highlights the need to consider second site mutations in genetic studies, even when working with well-established mutants.

A number of results support that JA/JA-Ile signaling has no important role in the response to photoperiod stress. First, the abolishment of JA synthesis did not reduce the stress phenotype. Lesion formation and reduction of photosynthetic activity in different mutants including *fad378*, *dde2/aos,* and *lox3 lox4* was similar as in WT and introgression of *dde2* into *ahk2 ahk3* did not alter the stronger stress response of the cytokinin receptor mutant. The induction of several stress response genes, correlating with the severity of the stress response, was not lowered in the presence of the *dde2* allele (Fig. 3C-F). These results not only exclude JA-Ile but also biologically active oxylipins/JAs other than JA-Ile (e.g. OPDA) as mediators of the response to photoperiod stress.

Analysis of JA signaling mutants also did not yield support for a strong role of the JA pathway in response to photoperiod stress. None of the JA signaling mutants (*coi1*, *myc234*, *ora59)* showed a strongly altered stress phenotype with respect to lesion formation and reduction of F_v_/F_m_, neither in WT nor in the sensitized CK-deficient background. This is important as it is different to the rescue of these traits caused by introgression of *jar1-1* (Nitschke et al. 2016; Supplemental Fig. 1). The results were partly different for the activation of stress response genes, which required COI1 and ORA59 in WT and COI1 but not ORA59 in *ahk2 ahk3* (Fig. 4) for a full response. This more complicated picture could be due to the fact that there are two separate signaling branches downstream of COI1, the MYC and the ERF1/ORA59 branch (Wasternack and Hause 2013). The pathway and the separate branches transmit signals of several effectors including JA but also other hormones (ABA for the MYC branch, ethylene for the ERF1/ORA59 branch) and light. In addition, these branches are not separate but interconnected (Wasternack and Hause 2013). Notably, the ABA concentration is increased after photoperiod stress (Nitschke et al. 2016) and various ethylene biosynthesis genes, including the S-adenosyl-L-methionine synthase (SAM) gene *SAM1,* the 1-aminocyclopropane-1-carboxylic acid (ACC) synthase genes *ACS6*, *ACS7* and *ACS8*, and ACC oxidase gene *ACO4*, are rapidly induced by photoperiod stress (Cortleven et al. 2022). It could thus be that these hormones contribute through the COI1/MYC/ORA59 pathway to the induction of *ZAT12* and *BAP1* expression, which respond to a variety of stresses (Davletova et al. 2005; van Buer et al., 2019). A more detailed investigation is needed to understand the role of this signaling pathway and additional signaling factors in the response to photoperiod stress.

Despite the functional irrelevance of JA/JA-Ile it was a possibility that JAR1 has an as yet unknown moonlighting function as has been described for the JA-Ile receptor COI1 (Ulrich et al. 2021) and other signaling proteins (Liu et al. 2022). Indeed, diverse roles in light signaling have been attributed to JAR1, which are realized through protein–protein interactions. *JAR1* is allelic to *FAR-RED INSENSITIVE 219* (*FIN219*), which was identified in a search for extragenic modifiers of a mutant allele of *CONSTITUTIVE PHOTOMORPHOGENIC 1* (*COP1*), which encodes an E3 ubiquitin-protein ligase regulating various aspects of light signaling (Hsieh et al. 2000). Physical interaction between JAR1/FIN219 and COP1 retained COP1 in the cytoplasm, thus protecting its target HY5 from degradation (Wang et al. 2011; Hsieh and Okamoto 2014). JAR1/FIN219 is also a positive regulator in phyA-mediated far-red (FR) light signaling (Hsieh et al. 2000; Chen et al. 2007), it is required for part of the transcriptional response to FR (Chen et al. 2015), it functions as a negative regulator in the shade avoidance response (Swain et al. 2017) and antagonizes the blue light receptor CRY1 by direct interaction (Chen et al. 2018). However, the option of a role of JAR1/FIN219 in response to photoperiod stress linked to its special roles in the response to light and different from the formation of JA-Ile was excluded essentially by the results of the genetic analysis. Segregation analysis showed clearly that the resistance to photoperiod stress could be genetically separated from the *jar1-1* mutant locus. This indicated that a different unlinked recessive mutation than *jar1-1* is responsible for the resistance phenotype.

Unfortunately, it has not been possible to identify the *SOPS* gene in the frame of this work. SOPS is clearly required for the formation of peroxides and the full activation of stress marker genes in response to photoperiod stress. Photoperiod stress induces an apoplastic oxidative burst, which is at least partly due to reduced catalase and enhanced peroxidase activity (Abuelsoud et al. 2020). It could be that the SOPS protein is directly or indirectly involved in regulating these enzymes to regulate the formation of apoplastic ROS. Xu et al. (2015) concluded based on the analysis of genome-wide transcriptional changes that several pathways regulate the response to apoplastic ROS via combinatorial or overlapping mechanisms. Therefore, SOPS might be part of the complex oxidative stress response network. Other candidates are genes involved in light perception or starch metabolism that render Arabidopsis Col-0 less sensitive to photoperiod stress when mutated (Bajaj Hengge et al. 2025). In any case, it should be noted that the photoperiod stress response did not disappear completely indicating that a SOPS-independent stress response pathway exists as well (Fig. 1).

There are a number of second site mutations reported to be responsible for part of the phenotype of well-studied and often-used mutants. Among these are a *max4* mutation in the chalcon synthase defective *tt4(2YY6)* mutant (Bennett et al. 2006), a *pen2* mutation in the JA signaling mutant *coi1-16* (Westphal et al. 2008), and an *are1* mutation in the ethylene mutant *ctr1-1* controlling part of the mutant´s root response (Shin et al. 2013). In the auxin mutant *abp1-5* an unknown *phyB* null allele is likely responsible for the long hypocotyl phenotype attributed previously to *abp1-5* (Enders et al. 2015). Recently, Yu et al. (2023) revealed that a mutation in the *COP1* gene caused the early flowering phenotype reported repeatedly for mutants of the JA signaling genes *MYC2* and *MYC3.* The discovery of second site mutations being responsible for part of the original phenotype is not limited to EMS mutagenesis but was also reported for T-DNA insertion lines, for example in *abp1-1* (Dai et al. 2015; Michalko et al. 2015) and *abi1-3* (Wu et al. 2015), as well as in *pif3-3* generated by fast neutrons (Ji et al. 2024). A proposal has been made regarding best practice for studying the effects of genetic variants, including the examination of multiple alleles whenever possible (Bergelson et al. 2016). Notably, *jar1-1* has been used in many laboratories and the question arises in how far phenotypes attributed to mutation of *JAR1* and thus to the action of JA/JA-Ile may be in fact due to the background mutation in the *SOPS* locus.

Taken together, despite the increase of JA/JA-Ile in response to an extended light period this hormone is not causally involved in establishing prominent phenotypic features of photoperiod stress. It is rather the increase in salicylic acid determining at least part of the stress output, including the production of peroxides and the induction of stress marker genes, with a central role for NPR1 and components of systemic acquired resistance (Cortleven et al. 2022; Roeber-Terstegen 2024).

## Supplementary Information

Below is the link to the electronic supplementary material.

Supplementary file1 (PDF 406 KB)

## Data Availability

All data needed to evaluate the conclusions in the paper are present in the paper and the supporting information.

## References

[CR1] Abuelsoud W, Cortleven A, Schmülling T (2020) Photoperiod stress induces an oxidative burst-like response and is associated with increased apoplastic peroxidase and decreased catalase activities. J Plant Physiol, 153252. 10.1016/j.jplph.2020.153252 32949889

[CR2] Arvidsson S, Kwasniewski M, Riaňo-Pachón DM, Müller-Roeber B (2008) Quantprime – a flexible tool for reliable high-throughput primer design for quantitative PCR. BMC Bioinformatics 9:465 18976492 10.1186/1471-2105-9-465 PMC2612009

[CR3] Bajaj Hengge I, Cortleven A, Schmülling T (2025) Plastid- and photoreceptor-dependent signaling is required for the response to photoperiod stress. J Plant Physiol 306:154429 10.1016/j.jplph.2025.154429 39892167

[CR5] Bennett T, Sieberer T, Willett B, Booker J, Luschnig C, Leyser O (2006) The *Arabidopsis MAX* pathway controls shoot branching by regulating auxin transport. Curr Biol 16:553–563 16546078 10.1016/j.cub.2006.01.058

[CR6] Bergelson J, Buckler ES, Ecker JR, Nordborg M, Weigel D (2016) A proposal regarding best practices for validating the identity of genetic stocks and the effects of genetic variants. Plant Cell 28:606–609 26956491 10.1105/tpc.15.00502 PMC4826003

[CR11] Chen IC, Huang IC, Liu MJ, Wang ZG, Chung SS, Hsieh HL (2007) Glutathione S-transferase interacting with far-red insensitive 219 is involved in phytochrome A-mediates signaling in Arabidopsis. Plant Physiol 143:1189–1202 17220357 10.1104/pp.106.094185 PMC1820923

[CR12] Chen HJ, Chen CL, Hsieh HL (2015) Far-red light-mediated seedling development in Arabidopsis involves FAR-RED INSENSITIVE 219/JASMONATE RESISTANT1-dependent and independent pathways. PLoS ONE 10:e0132723 26176841 10.1371/journal.pone.0132723 PMC4503420

[CR13] Chen HJ, Fu TY, Yang SL, Hsieh HL (2018) FIN219/JAR1 and cryptochrome1 antagonize each other to modulate photomorphogenesis under blue light in Arabidopsis. PLoS Genet 14:e1007248 29561841 10.1371/journal.pgen.1007248 PMC5880400

[CR14] Chini A, Fonseca S, Fernández G, Adie B, Chico JM, Lorenzo O, García-Casado G, López-Vidriero I, Lozano FM, Ponce MR, Micol JL, Solano R (2007) The JAZ family of repressors is the missing link in jasmonate signalling. Nature 448:666–671 17637675 10.1038/nature06006

[CR15] Choi J, Huh SU, Kojima M, Sakakibara H, Paek KH, Hwang I (2010) The cytokinin-activated transcription factor ARR2 promotes plant immunity via TGA3/NPR1-dependent salicylic acid signaling in *Arabidopsis*. Dev Cell 19:284–295 20708590 10.1016/j.devcel.2010.07.011

[CR16] Chung HS, Koo AJ, Gao X, Jayanty S, Thines B, Jones AD, Howe GA (2008) Regulation and function of *Arabidopsis JASMONATE ZIM*-domain genes in response to wounding and herbivory. Plant Physiol 146:952–964 18223147 10.1104/pp.107.115691 PMC2259048

[CR94] Cortleven A, Nitschke S, Klaumünzer M, Abdelgawad H, Asard H, Grimm B, Riefler M, Schmülling T (2014) A novel protective function for cytokinin in the light stress response is mediated by the Arabidopsis histidine kinase2 and Arabidopsis histidine kinase3 receptors. Plant Physiol 164:1470–1483 10.1104/pp.113.224667 PMC3938634 24424319

[CR18] Cortleven A, Leuendorf JE, Frank M, Pezzetta D, Bolt S, Schmülling T (2019) Cytokinin action in response to abiotic and biotic stresses in plants. Plant Cell Environ 42:998–1018 30488464 10.1111/pce.13494

[CR19] Cortleven A, Roeber VM, Frank M, Bertels J, Lortzing V, Beemster G, Schmülling T (2022) The transcriptomic landscape of the photoperiodic stress response in *Arabidopsis thaliana* resembles the response to pathogen infection. Front Plant Sci 13:838284 35646013 10.3389/fpls.2022.838284 PMC9134115

[CR21] Dai X, Zhang Y, Zhang D, Chen J, Gao X, Estelle M, Zhao Y (2015) Embryonic lethality of Arabidopsis *abp1-1* is caused by deletion of the adjacent *BSM* gene. Nat Plants 1:15183 27057346 10.1038/nplants.2015.183 PMC4821195

[CR22] Davletova S, Schlauch K, Coutu J, Mittler R (2005) The zinc-finger protein Zat12 plays a central role in reactive oxygen and abiotic stress signaling in Arabidopsis. Plant Physiol 139:847–856 16183833 10.1104/pp.105.068254 PMC1256000

[CR23] Enders TA, Oh S, Yang Z, Montgomery BL, Strader LC (2015) Genome sequencing of Arabidopsis *abp1-5* reveals second-site mutations that may affect phenotypes. Plant Cell 27:1820–1826 26106149 10.1105/tpc.15.00214 PMC4531353

[CR24] Fernandez-Calvo P, Chini A, Fernandez-Barbero G, Chico JM, Gimenez-Ibanez S, Geerinck J, Eeckhout D, Schweizer F, Godoy M, Franco-Zorrilla JM, Pauwels L, Witters E, Puga MI, Paz-Ares J, Goossens A, Reymond P, De Jaeger G, Solano R (2011) The arabidopsis bHLH transcription factors MYC3 and MYC4 are targets of JAZ repressors and act additively with MYC2 in the activation of jasmonate responses. Plant Cell 23:701–715 21335373 10.1105/tpc.110.080788 PMC3077776

[CR25] Frank M, Cortleven A, Novak O, Schmülling T (2020) Root-derived *trans*-zeatin cytokinin protects Arabidopsis plants against photoperiod stress. Plant Cell Environm 43:2637–2649 10.1111/pce.13860 32716064

[CR26] Frank M, Cortleven A, Schmülling T (2022) The response to photoperiod stress in *Arabidopsis thaliana* depends on auxin acting as an antagonist to the protectant cytokinin. Intern J Mol Sci 23:2936 10.3390/ijms23062936 PMC8955046 35328357

[CR28] Heyl A, Riefler M, Romanov GA, Schmülling T (2012) Properties, functions and evolution of cytokinin receptors. Eur J Cell Biol 91:246–256 21561682 10.1016/j.ejcb.2011.02.009

[CR29] Higuchi M, Pischke MS, Mähönen AP, Miyawaki K, Hashimoto Y, Seki M, Kobayashi M, Shinozaki K, Kato T, Tabata S, Helariutta Y, Sussman MR, Kakimoto T (2004) *In planta* functions of the *Arabidopsis* cytokinin receptor family. Proc Natl Acad Sci U S A 101:8821–8826 15166290 10.1073/pnas.0402887101 PMC423279

[CR30] Howe GA, Major IT, Koo AJ (2018) Modularity in jasmonate signaling for multistress resilience. Annu Rev Plant Biol 69:387–416 29539269 10.1146/annurev-arplant-042817-040047

[CR31] Hsieh HL, Okamoto H (2014) Molecular interaction of jasmonate and phytochrome A signaling. J Exp Bot 65:2847–2857 24868039 10.1093/jxb/eru230

[CR32] Hsieh HL, Okamoto H, Wang M, Ang LH, Matsui M, Goodman H, Deng XW (2000) FIN219, an auxin-regulated gene, defines a link between phytochrome A and the downstream regulator COP1 in light control of Arabidopsis development. Genes Dev 14:1958–1970 10921909 PMC316819

[CR34] Inoue T, Higuchi M, Hashimoto Y, Seki M, Kobayashi M, Kato T, Tabata S, Shinozaki K, Kakimoto T (2001) Identification of CRE1 as a cytokinin receptor from *Arabidopsis*. Nature 409:1060–1063 11234017 10.1038/35059117

[CR35] Jackson SD (2009) Plant responses to photoperiod. New Phytol 181:517–531 19154317 10.1111/j.1469-8137.2008.02681.x

[CR36] Ji Z, Belfield EJ, Li S, Fu X, Harberd NP (2024) Discovery of a second-site *nia2* mutation in the background of multiple Arabidopsis PIF-related mutants containing the *pif3-3* allele. New Phytol 241:17–23 37891447 10.1111/nph.19344 PMC10952432

[CR37] Kieber JJ, Schaller GE (2014) Cytokinins. Arabidopsis. Book 12:e0168 10.1199/tab.0168 PMC3894907 24465173

[CR38] Kusch S, Thiery S, Reinstädler A, Gruner K, Zienkiewicz K, Feussner I, Panstruga R (2019) Arabidopsis *mlo3* mutant plants exhibit spontaneous callose deposition and signs of early leaf senescence. Plant Mol Biol 101:21–40 31049793 10.1007/s11103-019-00877-z

[CR39] Liu D, He Z, Xiao Y, Zhang G, Cao S, Yin W, Qian Y, Yin Y, Zhang J, Chen S, Chu C, Tong H (2022) A cryptic inhibitor of cytokinin phosphorelay controls rice grain size. Mol Plant 15:293–307 34562665 10.1016/j.molp.2021.09.010

[CR40] McConn M, Browse J (1996) The critical requirement for linolenic acid is pollen development, not photosynthesis in an Arabidopsis mutant. Plant Cell 8:403–416 12239389 10.1105/tpc.8.3.403 PMC161109

[CR41] Michalko J, Dravecká M, Bollenbach T, Friml J (2015) Embryo-lethal phenotypes in early *abp1* mutants are due to disruption of the neighboring *BSM* gene. F1000Res. 4: 1104 10.12688/f1000research.7143.1 PMC4642851 26629335

[CR43] Mosblech A, Thurow C, Gatz C, Feussner I, Heilmann I (2011) Jasmonic acid perception by COI1 involves inositol polyphosphates in *Arabidopsis thaliana*. Plant J 65:949–957 21205029 10.1111/j.1365-313X.2011.04480.x

[CR44] Neter J, Kutner M, Nachtsheim C, Wasserman W (1996) Applied Linear Statistical Models. The McGraw-Hill Companies, Inc

[CR46] Nishimura C, Ohashi Y, Sato S, Kato T, Tabata S, Ueguchi C (2004) Histidine kinase homologs that act as cytokinin receptors possess overlapping functions in the regulation of shoot and root growth in *Arabidopsis*. Plant Cell 16:1365–1377 15155880 10.1105/tpc.021477 PMC490032

[CR47] Nitschke S, Cortleven A, Iven T, Feussner I, Havaux M, Riefler M, Schmülling T (2016) Circadian stress regimes affect the circadian clock and cause jasmonic acid dependent cell death in cytokinin-deficient Arabidopsis plants. Plant Cell 28:1616–1639 27354555 10.1105/tpc.16.00016 PMC4981127

[CR48] Nitschke S, Cortleven A, Schmülling T (2017) Novel stress in plants by altering the photoperiod. Trends Plant Sci 11:913–916 10.1016/j.tplants.2017.09.005 28970000

[CR52] Pauwels L, Barbero GF, Geerinck J, Tilleman S, Grunewald W, Pérez AC, Chico JM, Bossche RV, Sewell J, Gil E, Garcia-Casado G, Witters E, Inzé D, Long JA, De Jaeger G, Solano R, Goossens A (2010) NINJA connects the co-repressor TOPLESS to jasmonate signaling. Nature 464:788–791 20360743 10.1038/nature08854 PMC2849182

[CR53] Pré M, Atallah M, Champion A, De Vos M, Pieterse CM, Memelink J (2008) The AP2/ERF domain transcription factor ORA59 integrates jasmonic acid and ethylene signals in plant defense. Plant Physiol 147:347–357 10.1104/pp.108.117523 PMC2442530 18467450

[CR54] Riefler M, Novák O, Strnad M, Schmülling T (2006) *Arabidopsis* cytokinin receptor mutants reveal functions in shoot growth, leaf senescence, seed size, germination, root development, and cytokinin metabolism. Plant Cell 18:40–54 16361392 10.1105/tpc.105.037796 PMC1323483

[CR56] Roeber VM, Schmülling T, Cortleven A (2022) The photoperiod: handling and causing stress in plants. Front Plant Sci 12:781998 10.3389/fpls.2021.781988 PMC8821921 35145532

[CR57] Roeber-Terstegen VM (2024) Priming and memory by photoperiod stress in *Arabidopsis thaliana*. Doctoral thesis, Freie Universität Berlin.

[CR58] Sambrook J, Russell DW (2001) Molecular Cloning. 3rd ed., Cold Spring Harbor Laboratory Press.

[CR62] Sheard LB, Tan X, Mao H, Withers J, Ben-Nissan G, Hinds TR, Kobayashi Y, Hsu FF, Sharon M, Browse J, He SH, Rizo J, Howe GA, Zheng N (2010) Jasmonate perception by inositol-phosphate-potentiated COI1-JAZ co-receptor. Nature 468:400–405 20927106 10.1038/nature09430 PMC2988090

[CR63] Shin K, Lee RA, Lee I, Lee S, Park SK, Soh MS (2013) Genetic identification of a second modifier of ctr1-1 that controls ethylene-responsive and gravitropic root growth in *Arabidopsis thaliana*. Mol Cells 36:88–96 23740431 10.1007/s10059-013-0097-7 PMC3887932

[CR64] Staswick PE, Tiryaki I (2004) The oxylipin signal jasmonic acid is activated by an enzyme that conjugates it to isoleucine in Arabidopsis. Plant Cell 16:2117–2127 15258265 10.1105/tpc.104.023549 PMC519202

[CR65] Staswick PE, Su W, Howell SH (1992) Methyl jasmonate inhibition of root growth and induction of a leaf protein are decreased in an *Arabidopsis thaliana* mutant. Proc Natl Acad Sci USA 89:6837–6840 11607311 10.1073/pnas.89.15.6837 PMC49599

[CR67] Staswick PE, Tiryaki I, Rowe ML (2002) Jasmonate response locus *JAR1* and several related *Arabidopsis* genes encode enzymes of the firefly luciferase superfamily that show activity on jasmonic, salicylic, and indole-3-acetic acids in an assay for adenylation. Plant Cell 14:1405–1415 12084835 10.1105/tpc.000885 PMC150788

[CR68] Stinzi A, Browse J (2000) The Arabidopsis male-sterile mutant, opr3, lacks the 12-oxophytodienoic acid reductase required for jasmonate synthesis. Proc Natl Acad Sci USA 97:10625–10630 10973494 10.1073/pnas.190264497 PMC27075

[CR69] Suzuki T, Miwa K, Ishikawa K, Yamada H, Aiba H, Mizuno T (2001) The *Arabidopsis* sensor His-kinase, AHK4, can respond to cytokinins. Plant Cell Physiol 42:107–113 11230563 10.1093/pcp/pce037

[CR70] Swain S, Jiang HW, Hsieh HL (2017) FAR-RED INSENSITIVE 219/JAR1 contributes to shade avoidance responses of Arabidopsis seedlings by modulating key shade signaling components. Front Plant Sci 8:1901 29163619 10.3389/fpls.2017.01901 PMC5673645

[CR72] Thines B, Katsir L, Melotto M, Niu Y, Mandaokar A, Liu G, Nomura K, He SY, Howe GA, Browse J (2007) JAZ repressor proteins are targets of the SCF^COI1^ complex during jasmonate signalling. Nature 448:661–665 17637677 10.1038/nature05960

[CR74] Ulrich L, Schmitz J, Thurow C, Gatz C (2021) The jasmonoyl-isoleucine receptor CORONATINE INSENSITIVE1 suppresses defense gene expression in Arabidopsis roots independently of its ligand. Plant J 107:1119–1130 34145662 10.1111/tpj.15372

[CR75] van Buer J, Prescher A, Baier M (2019) Cold-priming of chloroplast ROS signalling is developmentally regulated and is locally controlled at the thylakoid membrane. Sci Rep 9:3022 30816299 10.1038/s41598-019-39838-3 PMC6395587

[CR77] Vandesompele J, De Preter K, Pattyn F, Poppe B, Van Roy N, De Paepe A, Speleman F (2002) Accurate normalization of real-time quantitative RT-PCR data by geometric averaging of multiple internal control genes. Genome Biol 3: research 0034 10.1186/gb-2002-3-7-research0034 PMC126239 12184808

[CR78] von Malek B, van der Graaff E, Schneitz K, Keller B (2002) The arabidopsis male-sterile mutant dde2-2 is defective in the ALLENE OXIDE SYNTHASE gene encoding one of the key enzymes of the jasmonic acid biosynthesis pathway. Planta 216:187–192 12430030 10.1007/s00425-002-0906-2

[CR79] Wallis JG, Browse J (2002) Mutants of Arabdidopsis reveal many roles for membrane lipids. Prog Lipid Res 41:254–278 11814526 10.1016/s0163-7827(01)00027-3

[CR80] Wang JG, Chen CH, Chien CT, Hsieh HL (2011) FAR-RED INSENSITIVE219 modulates CONSTUTITIVE PHOTOMORPHOGENIC1 activity via physical interaction to regulate hypocotyl elongation in Arabidopsis. Plant Physiol 156:631–646 21525334 10.1104/pp.111.177667 PMC3177264

[CR81] Wang L, Kim J, Somers DE (2013) Transcriptional corepressor TOPLESS complexes with pseudoresponse regulator proteins and histone deacetylases to regulate circadian transcription. Proc Natl Acad Sci USA 110:761–766 23267111 10.1073/pnas.1215010110 PMC3545823

[CR82] Wasternack C, Feussner I (2018) The oxylipin pathways: Biochemistry and function. Annu Rev Plant Biol 69:363–386 29166128 10.1146/annurev-arplant-042817-040440

[CR83] Wasternack C, Hause B (2013) Jasmonates: biosynthesis, perception, signal transduction and action in plant stress response, growth and development. An update to the 2007 review in Annals of Botany. Ann Bot 111:1021–1058 23558912 10.1093/aob/mct067 PMC3662512

[CR84] Wasternack C, Kombrink E (2010) Jasmonates: structural requirements for lipid-derived signals active in plant stress responses and development. ACS Chem Biol 5:63–77 20025249 10.1021/cb900269u

[CR85] Werner T, Schmülling T (2009) Cytokinin action in plant development. Curr Opin Plant Biol 12:527–538 19740698 10.1016/j.pbi.2009.07.002

[CR86] Werner T, Motyka V, Laucou V, Smets R, Van Onckelen H, Schmülling T (2003) Cytokinin-deficient transgenic *Arabidopsis* plants show multiple developmental alterations indicating opposite functions of cytokinins in the regulation of shoot and root meristem activity. Plant Cell 15:2532–2550 14555694 10.1105/tpc.014928 PMC280559

[CR87] Westphal L, Scheel D, Rosahl S (2008) The *coi1-16* mutant harbors a second-site mutation rendering PEN2 nonfunctional. Plant Cell 20:824–826 18430804 10.1105/tpc.107.056895 PMC2390735

[CR88] Wu Y, Li Y, Liu Y, Xie Q (2015) Cautionary notes on the usage of *abi1-2* and *abi1-3* mutants of Arabidopsis ABI1 for functional studies. Mol Plant 8:335–338 25624146 10.1016/j.molp.2014.10.007

[CR89] Xie DX, Feys BF, James S, Nieto-Rostro M, Turner JG (1998) *COI1*: an *Arabidopsis* gene required for jasmonate-regulated defense and fertility. Science 280:1091–1094 9582125 10.1126/science.280.5366.1091

[CR90] Xu E, Vaahtera L, Brosché M (2015) Roles of defense hormones in the regulation of ozone-induced changes in gene expression and cell death. Mol Plant 8:1776–1794 26348016 10.1016/j.molp.2015.08.008

[CR93] Yu D, Dong X, Zou K, Jiang XD, Sun YB, Min Z, Zhang LP, Cui H, Hu JY (2023) A hidden mutation in the seventh WD40-repeat of COP1 determines the early flowering trait in a set of Arabidopsis *myc* mutants. Plant Cell 35:345–350 36331342 10.1093/plcell/koac319 PMC9806556

