# Supplementary material for: An extragenic second-site mutation in the jar1-1 mutant suppresses the response to photoperiod stress independent of jasmonic acid
Source: Plant Mol Biol. 2025 Jun 29;115(4):79. doi: 10.1007/s11103-025-01602-9 (PMC12206676; doi:10.1007/s11103-025-01602-9)
Supplement: Supplementary file 1 — Supplementary file1 (PDF 406 KB) [file 11103_2025_1602_MOESM1_ESM.pdf]

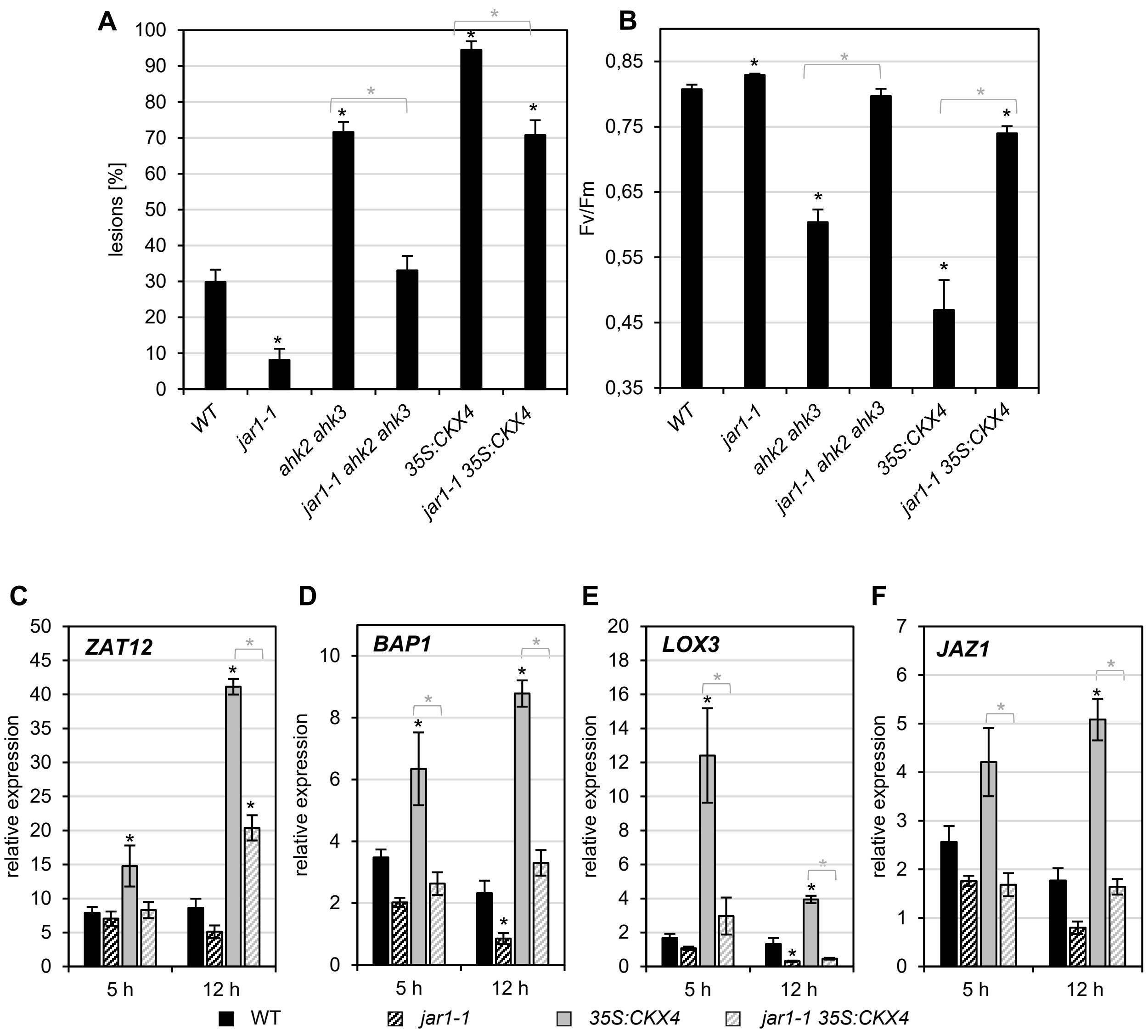


**Supplemental Fig. S1** Introgression of the *jar1-1* mutation into a cytokinin-deficient background causes a reduced induction of stress marker genes in response to photoperiod stress. Plants were grown under short day conditions for five weeks before exposure to a 32-hours light period (PL). A, Percentage of mature leaves with lesions ($n \geq 12$). B, PSII maximum quantum efficiency (Fv/Fm) in representative leaves measured around midday following PL treatment ($n \geq 12$). C-F, Transcript levels of oxidative stress marker genes *ZAT12* (C) and *BAP1* (D), JA biosynthesis gene *LOX3* (E) and JA signaling gene *JAZ1* (F) during the dark period at 5 h and 12 h after PL treatment. Transcript levels of WT 5 h control (data not shown) were set to 1. Data are mean values ± SE ($n = 4$). Asterisks indicate significant differences from WT (black) and between cytokinin-deficient plants in WT or *jar1-1* background (grey) ($p < 0.05$).

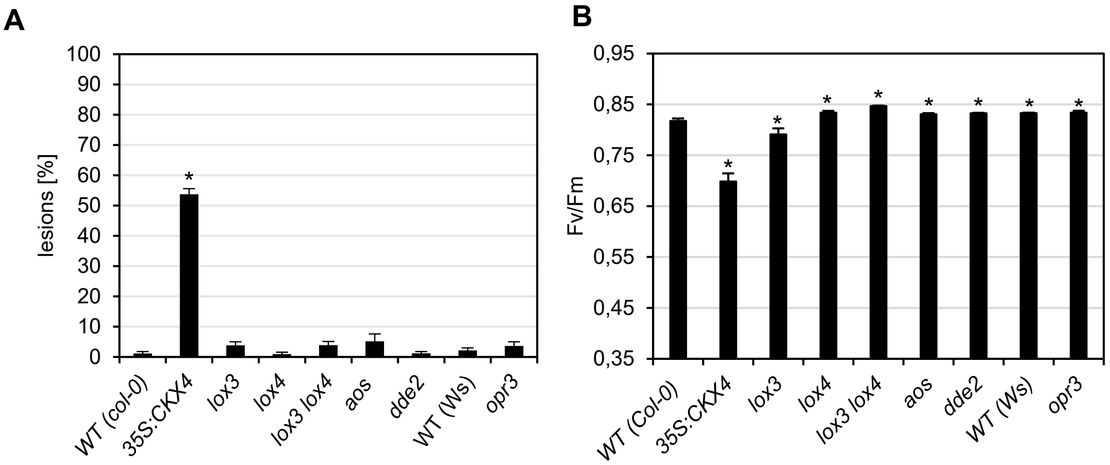


**Supplemental Fig. S2** The photoperiod stress response of JA biosynthesis mutants is comparable to wild type. Plants were grown under short day conditions for five weeks before exposure to a 32-h light period (PL). A, Percentage of mature leaves with lesions ($n \geq 12$). B, PSII maximum quantum efficiency (Fv/Fm) in representative leaves measured around midday following PL treatment ($n \geq 12$). Asterisks indicate significant differences from WT ($p < 0.05$).

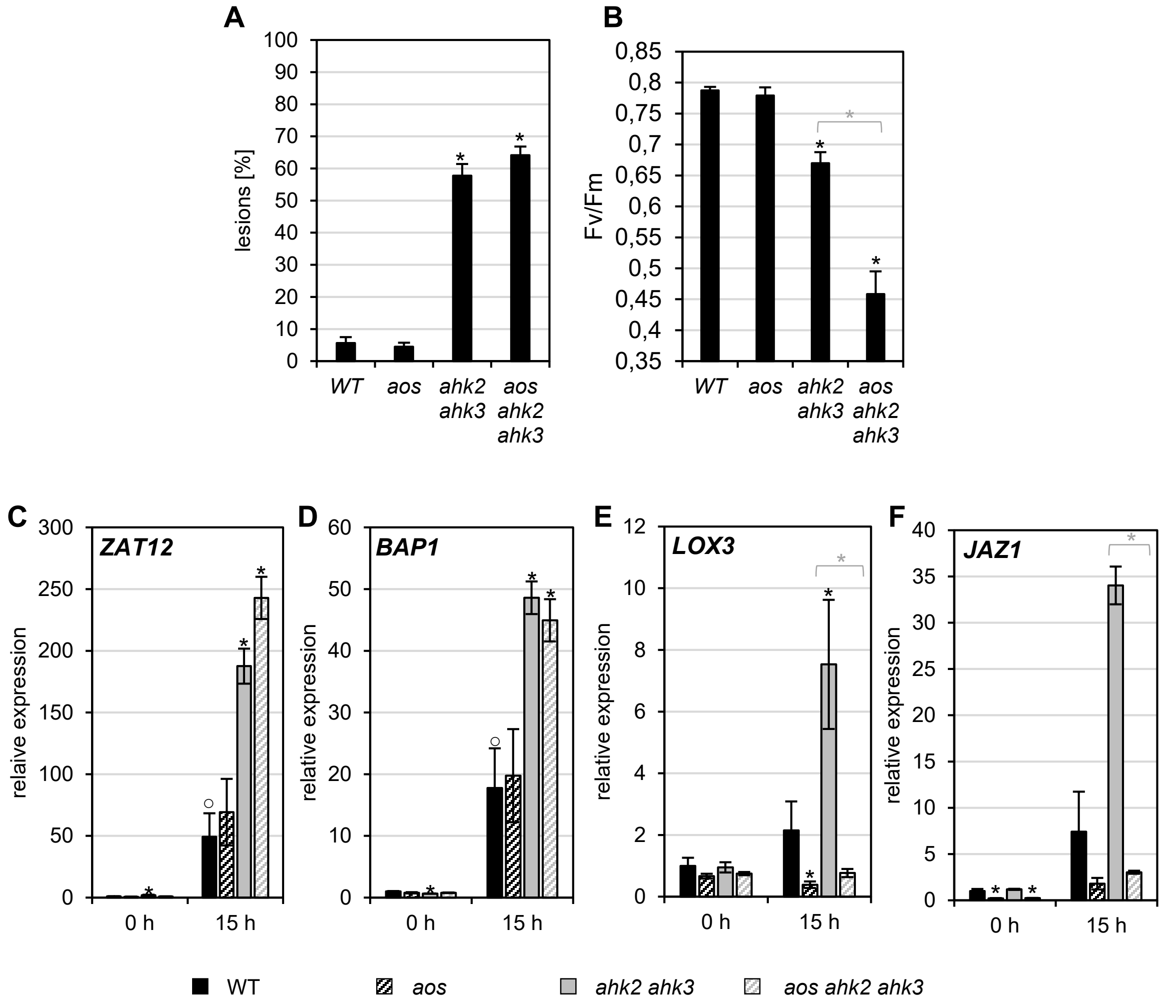


**Supplemental Fig. S3** A reduced JA biosynthesis does not rescue the photoperiod stress phenotype in cytokinin-deficient plants. Plants were grown under short day conditions for five weeks before exposure to a 32-h light period (PL). A, Percentage of mature leaves with lesions (n ≥ 12). B, PSII maximum quantum efficiency (Fv/Fm) in representative leaves (n ≥ 12) measured around midday following PL treatment. C-F, Transcript levels of oxidative stress marker genes *ZAT12* (C) and *BAP1* (D), JA biosynthesis gene *LOX3* (E) and JA signaling gene *JAZ1* (F) at the onset (0 h) and during the dark period at 15 h after PL treatment. Transcript levels of WT 0 h were set to 1. Data are mean values ± SE (n ≥ 3). Asterisks ($p \leq 0.05$) or + ($p \leq 0.084$) indicate significant differences from WT (black) and between cytokinin-deficient plants in WT or *aos* background (grey). Symbol ○ indicates significant differences between WT at 0 h and the other time points (for WT only) ($p \leq 0.05$).

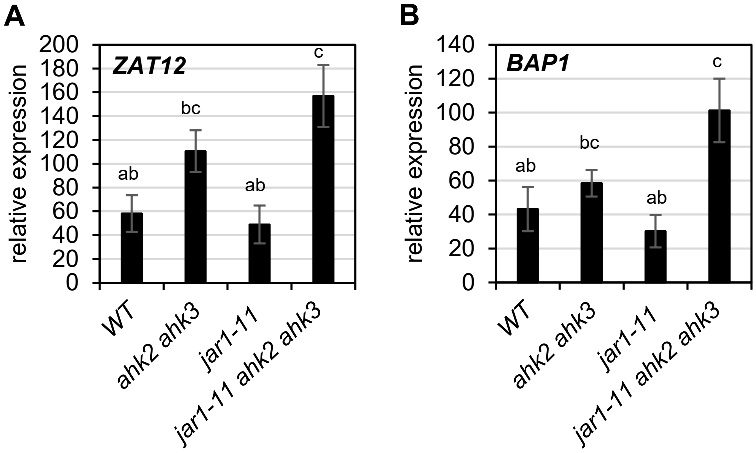


**Supplemental Fig. S4** Introgression of *jar1-11* in a cytokinin-deficient background does not alleviate the photoperiod stress syndrome. Plants were grown under short day conditions for five weeks before exposure to a 32-hours light period (PL). Sampling was done one hour before the light switches on. A-B, Transcript levels of oxidative stress marker genes *ZAT12* (A) and *BAP1* (B). Transcript levels of WT control were set to 1. Data are mean values ± SE (n = 4). Letters indicate different statistical groups (p-value ≤ 0.05).

**Supplemental Table S1** Sequences of primers used in this study.

**Quantitative real-time PCR primers**

| Gene Name | ATG number | Forward primer | Reverse primer |
|---|---|---|---|
| *BAP1* | AT3G61190 | CCAGAGATTACGGCGCGTGTT | TACAGACCCCAAACCGGAACTCC |
| *JAZ1* | AT1G19180 | CCCAACACCATTGACAGAAC | CTAAACCGAGCCACGACA |
| *LOX3* | AT1G17420 | ACGTTGTCGTACTGGTCGCC | GTCTCGTGGCACATACATAGGTAATG |
| *MCP2D* | AT1G79340 | AACCCGCTATGCAGACACACG | CAGTTGGTTTCCCCGCTGGA |
| *PP2A* | AT3G25800 | CCATTAGATCTTGTCTCTCTGCT | GACAAAACCCGTACCGAG |
| *ZAT12* | AT5G59820 | CGCTTTGTCGTCTGGATTG | AGCAGCCCCACTCTCGTT |
